# Decentralized Policy Coordination in Mobile Sensing with Consensual Communication

**DOI:** 10.3390/s22249584

**Published:** 2022-12-07

**Authors:** Bolei Zhang, Lifa Wu, Ilsun You

**Affiliations:** 1 School of Computer, Nanjing University of Posts and Telecommunications, Nanjing 210023, China; 2 State Key Laboratory for Novel Software Technology, Nanjing University, Nanjing 210046, China; 3 Department of Financial Information Security, Kookmin University, Seoul 02707, Republic of Korea

**Keywords:** mobile sensing, reinforcement learning, decentralized coordination, communication

## Abstract

In a typical mobile-sensing scenario, multiple autonomous vehicles cooperatively navigate to maximize the spatial–temporal coverage of the environment. However, as each vehicle can only make decentralized navigation decisions based on limited local observations, it is still a critical challenge to coordinate the vehicles for cooperation in an open, dynamic environment. In this paper, we propose a novel framework that incorporates consensual communication in multi-agent reinforcement learning for cooperative mobile sensing. At each step, the vehicles first learn to communicate with each other, and then, based on the received messages from others, navigate. Through communication, the decentralized vehicles can share information to break through the dilemma of local observation. Moreover, we utilize mutual information as a regularizer to promote consensus among the vehicles. The mutual information can enforce positive correlation between the navigation policy and the communication message, and therefore implicitly coordinate the decentralized policies. The convergence of this regularized algorithm can be proved theoretically under certain mild assumptions. In the experiments, we show that our algorithm is scalable and can converge very fast during training phase. It also outperforms other baselines significantly in the execution phase. The results validate that consensual communication plays very important role in coordinating the behaviors of decentralized vehicles.

## 1. Introduction

Over the past decade, the ubiquitous adoption of mobile vehicles has greatly enhanced the flexibility and convenience of environment sensing. When equipped with sensors, multiple vehicles can autonomously navigate to different locations to collect distributed environmental data. This paradigm, often referred to as mobile sensing, has attracted attention from a variety of disciplines, such as air quality sensing [1], traffic monitoring [2], fire detection [3], etc. For example, in a smart home, multiple devices (e.g., sweeping robots) can cooperate to sense the environment and perform related tasks [4], such as cleaning and tidying.

**Figure 1:**
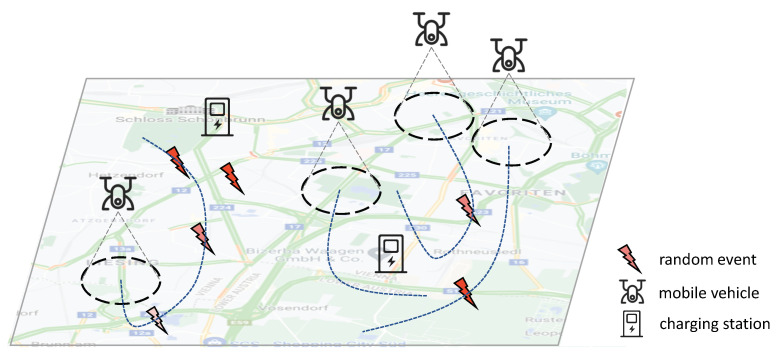
An illustration of the mobile sensing, where multiple vehicles cooperate to monitor the random events. The blue dash lines represent the moving trajectory of each vehicle. Events with a redder color imply higher intensities.

In a typical mobile sensing scenario, multiple events (e.g., fire, traffic jam, and pollution emission) may occur randomly and dynamically (depicted in Figure 1). Detecting such events in time is crucial for the mobile sensing application. However, since each vehicle can only observe the local environment within a limited radius, one central problem emerging is **how to navigate the decentralized vehicles to maximize the spatial–temporal coverage of the events**. As the vehicles need to make sequential navigation decisions, reinforcement learning (RL), in particular, multi-agent reinforcement learning (MARL) methods, have become a promising approach. RL methods can be model-free to optimize the navigation policies through exploration and exploitation. They are, therefore, applicable in different scenarios, even when the environmental model is not assumed [5,6].

Despite the progress made in recent years, one critical challenge that has been largely overlooked is the decentralized coordination of the vehicles. As illustrated in Figure 1, the events are mostly distributed at the left and right sides of the map. It could be better if one of the right vehicles moves to the left area for sensing. However, without coordination, the right vehicles may compete to sense nearby events, leading to wasted sensing efforts. One possible direction to tackle this challenge is to use a centralized controller that manages the policies of all vehicles. However, centralized approaches may face the problem of “single point of failure” and low scalability.

To navigate multiple vehicles in an open, dynamic environment, we adopt the MARL as the basic solution. However, in the execution phase, the vehicles may still have uncoordinated behaviors due to the lack of common consensus [7,8,9]. Inspired by the recent advances of learning to communicate [10,11], we can also introduce the communication mechanism in the cooperative navigation. On one hand, the common signal can provide global information from all the vehicles. On the other hand, the other vehicles’ moving actions can also be inferred if there is positive correlation with the signal.

**Our Method** In this paper, we consider the decentralized management of the mobile vehicles, and introduce a communication-based framework to coordinate the behaviors of the vehicles. At each step before moving, the vehicles should first broadcast communication messages to others to share information. Afterwards, when receiving the communication messages from others, each vehicle can be conditioned on the received messages to take navigation actions. By adopting this communication framework, the vehicles can share information with each other to break through the dilemma of local observation. In particular, the communication message is also learned via reinforcement learning with the aim to maximize the spatial–temporal coverage of the events. This learning to communicate framework is flexible, and can be applicable in different dynamic environments.

One major concern in the communication framework is that the vehicles may simply ignore the communication message and focus only on local observations. To deal with this problem, in this paper, we try to **maximize the mutual information between the received messages and the vehicles’ navigation policies**. By maximizing this term, the mobile vehicles can correlate their policies with the received messages. Intuitively, a positive correlation implies that other vehicles’ policies can be inferred based on the received message. Therefore, the vehicles can achieve consensus implicitly. Theoretical analysis shows that this regularized algorithm can converge to equilibrium points under certain mild conditions.

In the experiment part, we implement and evaluate the proposed algorithm in a simulation environment built from a real-world data set. We first validate the decentralized algorithm in both the training and execution phases. The results show that the consensual communication framework can successfully coordinate the behaviors of the decentralized vehicles. The mutual information term plays an important role in the coordination. Our method can also adapt to multiple scenarios with different hyper-parameters. In different settings, our algorithm can consistently outperform other baselines. Our work can be widely adopted in different fields, such as smart homes, smart city, agriculture, etc.

### 1.1. Contributions

Our key contributions are listed as follows:

- We model the mobile sensing problem as a decentralized sequential optimization problem, where the vehicles navigate to maximize the spatial-temporal coverage of the events in the environment.
- A communication framework is proposed for cooperative navigation. In particular, the communication protocol is learned by model-free reinforcement learning methods.
- We explicitly correlate the vehicles moving policies with the communication messages to promote coordination. The regularized algorithm can be proved to converge to equilibrium points under certain mild assumptions.
- Extensive experiments are conducted to show the effectiveness of our approach.

### 1.2. Organizations

The rest of the paper is organized as follows. We first introduce the related work in Section 2. Next, we formulate the system model and the optimization objective in Section 3. Section 4.1 presents the framework of learning to communicate. We then present how to enforce positive communication in Section 5. Evaluation is given in Section 6. We conclude the paper in Section 7.

## 2. Related Work

In this section, we first introduce the recent advances in reinforcement learning, which is the main technical solution in this work. Next, we will review the related works of mobile sensing, with a focus on how to navigate the mobile vehicles in the environment to maximize the event coverage.

### 2.1. Reinforcement Learning

Reinforcement learning (RL) has achieved great success in wide areas, such as Game of Go [12], Atari [13], Starcraft [8], etc. The problem of RL can generally be modeled as a Markov decision process (MDP) 〈S,A,T,R,γ〉, where S is the state space, A is the action space, and T:S×A→S is the transition model for generating the next state. R:S×A→R is the reward function. γ∈(0,1] is a discount factor. At each step *t*, when an agent observes the state $s^{t}\in S$ and executes an action $a^{t}\in A$, it will then be transitioned into a new state $s^{t+1}$ and receive an immediate reward $r^{t}$, with probability $p(s^{t+1},r^{t}|s^{t},a^{t})\in T$. Let $R^{t}$ denote the cumulative return at time *t*. In an infinite horizon MDP, the cumulative return can be represented as

(1) $$R^{t}=\lim_{h\to \infty }\frac{1}{h}\sum_{t^{\prime }=t}^{h}E\left[r^{t^{\prime }}\right]$$

The goal of reinforcement learning is to find the optimal policy $\mu^{*}$ to maximize the return: $\mu^{*}=\arg\max_{\mu }E_{\mu }\left[R^{0}\right]$, where policy $\mu (a_{t}|s_{t})$ is a function which maps the state $s_{t}$ to a distribution of actions $a_{t}$. MDP has the property of the Bellman equality:

(2) $$Q\left(s^{t},a^{t}\right)=r^{t}+\gamma \sum_{s^{t+1}}p\left(s^{t+1}|s^{t},a^{t}\right)v\left(s^{t+1}\right)$$

where $Q\left(s,a\right)=E\left[R^{t}|s^{t}=s,a^{t}=a\right]$ is the state-action value function and $v\left(s\right)=E\left[R^{t}|s^{t}=s\right]$ is the value function of state *s*.

The process of RL can be generally divided into training and execution phases. In the training phase, the RL agent uses exploration and exploitation in the environment to optimize the policy. While in the execution phase, the agent will fix the policy parameters in the environment. In this paper, as the vehicles need to move in a continuous space, we focus on DDPG [14,15], which can generate continuous actions. In DDPG, there is a critic function to evaluate the state-action value by following a deterministic policy μ as $Q^{\mu }\left(s^{t},a^{t}\right)$, and an actor function which maps the state $s^{t}$ to a deterministic action, $a^{t}=\mu \left(s^{t}\right)$.

Recently, multi-agent reinforcement learning (MARL) has also been a hot research topic. MARL models the environment as a decentralized partially observable Markov decision process (Dec-POMDP) [8,9] as a tuple $\langle S,T,A,R,O,I\rangle$, where O is the set of local observations and I is the set of agents. The agents that make decisions are based on the observations. Let $o_{i}^{t}\in O\subseteq S$ be the local observation of agent *i* at step *t*. Each agent *i* can choose an action $a_{i}^{t}\in A$, forming a joint action $a^{t}\in A^{n}$, and transition to the next state $s^{t+1}\in S$ according to the function $p(s^{t+1},r^{t}|s^{t},a^{t})\in T$, where the reward function $r^{t}\in R$ is shared by all the agents.

To optimize the policies of the agents in MARL, previous works, such as COMA [8], MADDPG [9], QMIX [7], etc., mainly adopted the “centralized training, decentralized execution” (CTDE) mechanism: during training, global state information can be used to train the policy network; and during execution, the agents can only condition on local observations. In the execution phase, the agents could still change their policies dynamically, leading to incoordination of the decentralized policies. However, we address that such a CTDE mechanism may not be applicable in decentralized environments where the agents can only be trained separately. Recent works are considering methods of learning to communicate [16,17,18,19], where the communication policy is learned via RL. We will also adopt this mechanism in our work. In comparison to previous works [16,17,19] that mostly use lazy communication, we propose to enforce positive communication so that the messages can be utilized more efficiently. Moreover, most of previous works only used ungrounded, cheap talk for communication [10]. We address that such cheap talk communication may not be effective in coordination.

### 2.2. Mobile Sensing

Mobile sensing has been extensively studied with the emergency of autonomous vehicles. One of the main problem is maximizing the coverage of events in the environment. Earlier works mostly assumed that the environment model is a prior and proposed combinatorial optimization method. For example, Karaliopoulos et al. [20] modeled the problem as a cover problem and proposed the approximation ratio algorithm. Hu et al. [21] also proposed mobile sensing methods with spatial–temporal awareness. The paper adopted a combinatorial pinning zero-determinant (ZD) strategy to find a cost-efficient mobile sensing strategy. In comparison, our work addresses the dynamics of the environment, and the coordinated policies of different mobile users are learned via repeated interactions.

As the users make independent decisions, decentralized algorithms based on game theory were also considered. Rahili et al. [22] designed a rule-based communication protocol in which agents can communicate with local neighbors and use their local information make decisions. Esch et al. [23] depicted a distributed algorithm where the agents can communicate with one another wirelessly within a fixed communication radius. Li et al. [24] modeled the mobile crowdsourcing as a Stackelberge game, and proposed a three-party evolutionary game model for task allocation. However, most previous methods are hard to generalize to unseen scenarios. In an open environment, it is critical for the agents to adapt to dynamic environment events. Data privacy is also important in mobile sensing and has been a hot research topic very recently [25,26,27,28,29]. In comparison, we focus more on the navigation of the mobile vehicles instead of the data-collecting process.

When the environment model is unknown, machine learning approaches attract attention [30,31]. In particular, as the environment is often dynamic [32,33], online learning or RL-based algorithms are widely considered, which are sequential and model-free. An et al. [34] adopted the multi-armed bandits method to select users to improve service quality. However, bandit algorithms neglect the sequential behavior of agents and may not be feasible for mobile sensing problems. As RL uses deep learning to extract the representation of the environment for exploration and exploitation, it can be naturally applicable in the dynamic environment. For example, Zhang et al. [35] adopted RL for a coarse-to-fine deep scheme to address the aspect ratio variation in UAV tracking. Liu et al. [36,37] used deep RL for high quality data collection. The main idea is to employ multiple mobile vehicles to schedule their paths independently to maximize the coverage of distributed POIs (point of interests). Zeng et al. [38] divided the problem into four sub-optimal problems, and used an iterative algorithm solve the optimal problem. Liu et al. [5] proposed a multi-UAV mobile sensing framework based on multi-agent reinforcement learning (MARL), and utilized “centralized training decentralized execution” (CTDE) for cooperation. Wei et al. [6] considered the multi-robot informative path planning problem and proposed independent learning through credit assignment for cooperative sensing. Samir et al. [39] leveraged unmanned aerial vehicles (UAVs) for mobile sensing and proposed an RL approach to maximize the sensing coverage. A major challenge in these works is to coordinate the policies of different mobile vehicles for cooperation. While most previous works implicitly learn the cooperation policies for each agent, in our work, we addressed that coordination is crucial and explicitly proposed policy coordination methods based on consensual communication.

## 3. System Model

In this paper, we consider a mobile sensing problem where a set of mobile vehicles I={1,2,…,N} cooperate to maximize the spatial–temporal sensing coverage of the events in the environment. Suppose the time horizon is divided into infinite discrete intervals as {0,1,2,…,∞}. At each interval *t*, each vehicle i∈I at position $\left(x_{i}^{t},y_{i}^{t}\right)$ need to decide the moving action $a_{i}^{t}$, which can be represented as a tuple of speed $\nu_{i}^{t}\in \left[0,S_{\max}\right]$ and angle $\phi_{i}^{t}\in \left[0,2\pi \right)$, i.e., $a_{i}^{t}=\left(\nu_{i}^{t},\phi_{i}^{t}\right)$. After moving, the new position will be $\left(x_{i}^{t}+\nu_{i}^{t}\sin\phi_{i}^{t},y_{i}^{t}+\nu_{i}^{t}\cos\phi_{i}^{t}\right)$. Meanwhile, vehicle *i* is associated with a battery capacity $b_{i}^{t}\in \left[0,b_{\max}\right]$. The battery has a consumption rate $\Delta_{i}^{t}$ that is linear with the vehicle speed, i.e., $\Delta_{i}^{t}=\beta \nu_{i}^{t}+\Delta_{0}$, where β is a coefficient and $\Delta_{0}$ is a constant intrinsic battery consumption. The battery capacity will be updated as $b_{i}^{t+1}=b_{i}^{t}-\Delta_{i}^{t}$ each time. To avoid running out of power, the vehicles should regularly move to the charging station, in which the battery will be recharged for a fixed number of units $b_{0}$ at each interval.

In the environment, random events may happen at different positions with time-varying intensities. Let E be the set of events. We use $\tau_{e}^{t}$, e∈E to represent the event intensity of *e* at step *t*. The event *e* at position $\left(x_{e}^{t},y_{e}^{t}\right)$ is sensed/covered by vehicle *i* if it is within a limited radius of *i*. Let ${𝟙}_{ie}^{t}$ be an indicator function to represent if the event is covered by vehicle *i*:

(3) $${𝟙}_{ie}^{t}=\left\{\begin{array}{cc} 1,\;\;\; & \;\mathrm{if}\;\sqrt{{\left(x_{i}^{t}-x_{e}^{t}\right)}^{2}+{\left(y_{i}^{t}-y_{e}^{t}\right)}^{2}}\leq l_{i},\\ 0,\;\;\; & \;o.w. \end{array}\right.$$

where $l_{i}$ is the sensing radius of vehicle *i*. The benefit will be $\tau_{e}^{t}$ if the event *e* is covered by at least one of the mobile vehicles. Note that if multiple vehicles cover the same event *e* simultaneously, the benefit is still $\tau_{e}^{t}$. Therefore, the vehicles should cooperate to avoid repeatedly sensing the same event. We use ${𝟙}_{e}^{t}$ as an indicator function that the event *e* is covered by at least one vehicle at interval *t*, i.e., ${𝟙}_{e}^{t}=\max\left\{{𝟙}_{1e}^{t},{𝟙}_{2e}^{t},\ldots ,{𝟙}_{Ne}^{t}\right\}$. The problem can then be formulated as finding the joint moving policies for the vehicles, so that the cumulative sensing coverage of the events is maximized:

(4) $$\begin{array}{cc} \; & \max\sum_{t=0}^{\infty }\sum_{e\in E}\tau_{e}^{t}{𝟙}_{e}^{t}\\ \; & s.t.\;b_{i}^{t}\geq 0,\forall i\in I,\forall t\in \left\{0,1,2,\ldots \infty \right\}. \end{array}$$

The inequality constraint in the objective means that the mobile vehicles could no longer move or sense when running out of battery. According to the objective, the vehicles need to make sequential navigation decisions to cover the dynamic events. However, as the vehicles make decentralized decisions, it could be difficult for the vehicles to acknowledge others’ observations or intentions. This brings the dilemma of local observation and will be the main focus of this paper. Table 1 summarizes the key parameters in this paper.

## 4. Learning to Communicate

To break through the dilemma of local observation, in this section, we first formulate the problem as a Markov game. Then we formally introduce the communication framework, where the vehicles can share information with each other. Finally, we will show how to optimize the moving policies of each vehicle under this framework.

### 4.1. Mobile Sensing as a Markov Game

According to the system model, we can formulate the mobile sensing problem as a Dec-POMDP with tuples of $\langle S,O,A,T,R,I\rangle$, where the set of agents I represent the mobile vehicles. Now we give the definitions of other elements as follows:

- *State*: In the mobile sensing problem, at each interval *t*, the system state $s^{t}\in S$ includes the global information of the environment.
- *Observation*: In the environment, each vehicle *i* can only partially observe the state. The observation $o_{i}^{t}\in O$ is the subset of the environment state: $o_{i}^{t}\subseteq s^{t}$. We assume that each vehicle can observe the environment information within the sensing radius $l_{i}$, including its own position, last moving action, remaining battery capacity and sensed events.
- *Action*: The action of the mobile vehicle *i* is a continuous tuple $a_{i}=\left(\nu_{i}^{t},\phi_{i}^{t}\right)\in A_{i}$, where $\nu_{i}^{t}$ is the speed, and $\phi_{i}^{t}$ represents the moving angle. At each interval, all the vehicles will take the moving action to form a joint action $a^{t}=\left(a_{1}^{t},a_{2}^{t},\ldots ,a_{N}^{t}\right)$.
- *Transition*: Given the joint actions of the vehicles, the environment will transit to the next state $s^{t+1}$ according to the transition function:
  (5) $$p\left(s^{t+1}|s^{t},a^{t}\right):S\times A_{1}\times \ldots \times A_{N}\to S$$
  Note that this function is not known to be used, and can only be inferred through repeated interaction with the environment.
- *Reward*: As the mobile vehicles cooperate to maximize the spatial–temporal coverage of the environment, we define a global reward as the sensed events intensities:
  (6) $$r^{t}=\sum_{e\in E}\tau_{e}^{t}{𝟙}_{e}^{t}$$
  However, for each vehicle, it is intricate to infer its contribution to the global reward. Therefore, we decompose the reward function and define the individual reward for each vehicle *i* as
  (7) $$r_{i}^{t}=\sum_{e\in E,{𝟙}_{ie}^{t}=1}\frac{\tau_{e}^{t}{𝟙}_{ie}^{t}}{\sum_{k\in I}{𝟙}_{ke}^{t}}$$
  The reward function indicates that the reward of sensing event *e* is averaged by the number of vehicles that cover *e* at this step. It is obvious to see that $r^{t}=\sum_{i\in I}r_{i}^{t}$. To take the battery capacity into account, we relax the constraint in Equation (4) with an additional term *c* when the vehicle runs out of battery power. The vehicles will receive this penalty when the capacity is below zero, i.e., $c(b_{i}^{t})=c$ if $b_{i}^{t}<0$; otherwise, $c(b_{i}^{t})=0$. The value of *c* balances the preference between sensing a reward and penalty of battery loss. The relaxed version of the reward can be formulated as
  (8) $$r_{i}^{t}=\sum_{e\in E,{𝟙}_{ie}^{t}=1}\frac{\tau_{e}^{t}{𝟙}_{ie}^{t}}{\sum_{k\in I}{𝟙}_{ke}^{t}}-c\left(b_{i}^{t}\right)$$

### 4.2. The Communication Framework

**Figure 2:**
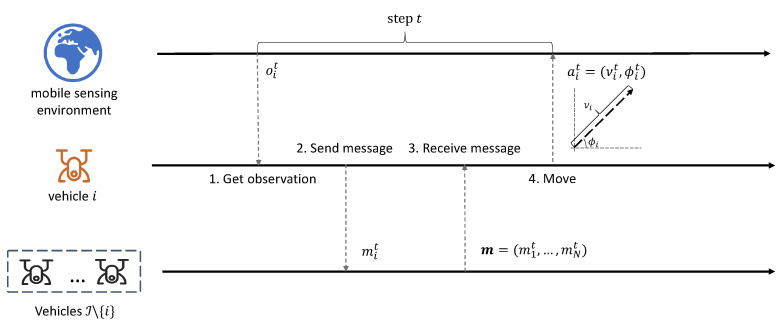
An illustration of the procedure. With local observation, the vehicles first exchange their messages by broadcasting. Afterwards, they make the moving decision based on the received message and local observation. This figure shows the communication process of the ego vehicle *i* in a single step *t*: The vehicle first obtains the observation $o_{i}^{t}$ in the environment. The vehicle then broadcasts a message $m_{i}^{t}$ to other vehicles based on the observation. The messages $\left(m_{1}^{t},\ldots ,m_{N}^{t}\right)$ from other vehicles will also be aggregated as part of the observation. Vehicle *i* will finally make the moving decision $a_{i}^{t}$ based on the environment observation and the aggregated message.

As the vehicles only have limited observation, we introduce a communication framework to share information among the vehicles. Figure 2 presents an illustration of the communication procedure. We now separately describe how to broadcast and receive the messages.

**Communication Broadcasting** As presented in Figure 2, at each step *t* before moving, each vehicle *i* first broadcasts a message $m_{i}^{t}$ to other vehicles. When broadcasting the message, an intuitive idea is to send the observation $o_{i}^{t}$ and the intended action $a_{i}^{t}$ to other vehicles. However, this is not possible since the vehicle will also be conditioned on the received messages from others to take action $a_{i}^{t}$. Moreover, the dimensions of the observation may be large with high overhead. Instead, we introduce the mechanism of **learning to communicate**. Suppose vehicle *i* uses a communication policy network $\mu_{i}^{m}\left(o_{i}^{t}\right)$ parameterized by $\theta_{i}^{m}$ to output the message content $m_{i}$, which can be a fixed-size continuous vector. In particular, the communication policy network can be optimized via the RL-based algorithm, where the goal is the long-term cumulative sensing coverage of the events. By learning to communicate, the vehicles can encode the observations and intentions into a compact embedding, which significantly reduces the transmission cost. Moreover, it can be flexible to deal with different scenarios and environments. More details on how to optimize the communication policy network will be introduced in Section 4.3.

**Communication Receiving** After broadcasting, each vehicle can also receive the messages from other vehicles: $m^{t}=\left(m_{1}^{t},m_{2}^{t},\ldots ,m_{N}^{t}\right)$. The messages can be aggregated with different operators, such as mean, max, or neural networks, such as recurrent neural networks (RNN). The aggregated message can be represented as $m_{g}=\mathrm{AGG}\left(m^{t}\right)$, where AGG is the aggregator of the received messages. Suppose the moving policy of vehicle is represented as $\mu_{i}\left(\cdot \right)$. It can be formulated as conditioning on the local observation and received messages for moving: $a_{i}^{t}=\mu_{i}^{t}\left(o_{i}^{t},m_{g}\right)$.

### 4.3. Policy Optimization

With the communication framework, we can now optimize the moving policy networks $\mu_{i}\left(\cdot \right)$ and communication policy networks $\mu_{i}^{m}\left(\cdot \right)$ for each vehicle i∈I. As the moving action of each vehicle is a continuous vector, we use DDPG for policy optimization. Let $Q_{i}\left(\cdot \right)$ be the action value function (critic) parameterized by $\theta_{i}^{Q}$. (We temporarily abbreviate the time indicator *t*. The sign ${}^{-}$ indicates t−1 and ${}^{\prime }$ indicates t+1.) The policy functions $\mu_{i}^{m}\left(\cdot \right)$, $\mu_{i}\left(\cdot \right)$ and the critic function $Q_{i}\left(\cdot \right)$ can all be implemented with neural networks. The parameters $\theta_{i}$ of the moving policy network $\mu_{i}\left(\cdot \right)$ can be updated according to the deterministic policy gradient theorem [14]:

(9) $$\nabla_{\theta_{i}}J\left(\mu_{i}\right)=E_{o_{i},m,a_{i}∼D}\left[\nabla_{\theta_{i}}\mu_{i}\left(o_{i},m_{g}\right)\nabla_{a_{i}}Q_{i}\left(o_{i},a_{i},m_{g}\right){|}_{a_{i}=\mu_{i}\left(o_{i},m_{g}\right)}\right]$$

where J(·) is the return of the policy and D is the set of historical data samples. Similarly, we can also update the parameters of the communication policy network $\mu_{i}^{m}$ as

(10) $$\nabla_{\theta_{i}^{m}}J\left(\mu_{i}^{m}\right)=E_{o_{i},m,a_{i}∼D}\left[\nabla_{\theta_{i}^{m}}\mu_{i}^{m}\left(o_{i}\right)\nabla_{m_{i}}Q_{i}\left(o_{i},a_{i},m_{g}\right){|}_{m_{i}=\mu_{i}^{m}\left(o_{i}\right)}\right]$$

where $\theta_{i}^{m}$ represents the parameters of the communication policy network. The action value network can be updated by minimizing the temporal difference (TD) error:

(11) $$\begin{array}{cc} L(\theta_{i}^{Q})= & E_{o_{i},m_{g},a_{i},r_{i},o_{i}^{\prime },m_{g}^{\prime }}[Q_{i}\left(o_{i},a_{i},m_{g}\right)-\\ \; & {\left(r_{i}+\gamma Q_{i}\left(o_{i}^{\prime },a_{i}^{\prime },m_{g}^{\prime }\right){|}_{m_{i}^{\prime }=\mu_{i}^{m}\left(o_{i}^{\prime }\right),a_{i}^{\prime }=\mu_{i}\left(o_{i}^{\prime },m_{g}^{\prime }\right)}\right]}^{2} \end{array}$$

According to the above formulations, we can update the parameters of the policy networks and action value networks simultaneously. Compared to the CTDE framework, which requires centralized training, in our framework, the networks can be optimized independently based on the local observation and communication messages. Therefore, this framework can be applicable in decentralized training scenarios.

## 5. Consensual Communication

By learning to communicate, the mobile vehicles can share local information with each other. However, previous works have shown that selfish agents do not learn to use this type of ungrounded, cheap talk communication channel effectively [11]. In this section, we first try to enforce the mobile vehicles to have **consensual communication**, i.e., the communication will indeed influence the vehicles’ behaviors. Next, we show that the algorithm can converge under the communication framework.

### 5.1. Mutual Information for Consensual Communication

To enforce positive communication, we maximize the **mutual information** between the moving policy $\mu_{i}$ and the aggregated message from *i*’s neighbors: $m_{g}$. Intuitively, by maximizing the mutual information, the vehicle can correlate its moving policy with the messages from neighbors. This can also be regarded as reducing the uncertainty of vehicles’ moving policy after receiving the messages. Formally, we augment the reward function as follows:

(12) $${\hat{r}}_{i}=\left(1-\rho \right)r_{i}+\rho I\left(\mu_{i};m_{g}\right)$$

where ρ∈[0,1] is a hyper-parameter that controls the importance of the mutual information term $I(\mu_{i};m_{g})$. The mutual information item can be expressed in terms of entropy and conditional entropy:

(13) $$\begin{array}{cc} I(\mu_{i};m_{g}) & =H\left(m_{g}\right)-H\left(m_{g}|\mu_{i}\right)\\ \; & =H\left(\mu_{i}\right)-H\left(\mu_{i}|m_{g}\right) \end{array}$$

where H(·) is the entropy function. The mutual information will become zero if the communication message does not influence the moving policy. In this case, $H(m_{g})$ equals $H(m_{g}|\mu_{i})$. Maximizing the mutual information indicates that *we enforce all the vehicles to correlate their policies with the message*. Thus, the vehicles can infer other neighbors’ behaviors by acknowledging the broadcast message, which implicitly promotes coordination among the vehicles. However, directly maximizing the MI is intractable. We instead introduce the variational distribution $q(m_{g}|\mu_{i})$ as a proxy for the posterior over $\mu_{i}$. Learning a neural network to predict the messages based on the policy $\mu_{i}$ provides a lower bound on MI:

(14) $$\begin{array}{cc} \; & I\left(\mu_{i};m_{g}\right)=H\left(m_{g}\right)-H\left(m_{g}|\mu_{i}\right)\\ \; & =H\left(m_{g}\right)+E_{m_{g},\mu_{i}}\left[\log q\left(m_{g}|\mu_{i}\right)\right]\\ \; & \;\;-E_{m_{g}}\left[D_{KL}\left(p\left(m_{g}|\mu_{i}\right)||q\left(m_{g}|\mu_{i}\right)\right)\right]\\ \; & \geq H\left(m_{g}\right)+E_{\mu_{i},m_{g}}[\log\left(q\left(m_{g}|\mu_{i}\right)\right] \end{array}$$

where $D_{KL}$ is the KL divergence between two probabilities. The establishment of inequality is because the KL-divergence distance is non-negative. In practice, as the policy $\mu_{i}$ is a network, we use historical observation–action trajectories $traj_{i}$ to represent the policy.

**Figure 3:**
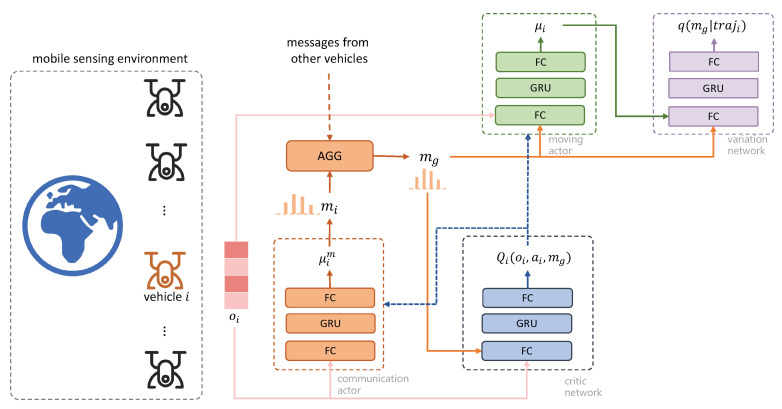
The network structure of policy coordination with consensual communication. FC represents fully connected layer, and GRU represents gated recurrent unit layer. AGG is the aggregator operator for the received messages. According to the network structure, each mobile vehicle needs to maintain 4 networks: moving actor network (the green part), critic network (the blue part), communication actor network (the orange part) and the variation network (the purple part).

The network structure of our framework is presented in Figure 3. For each vehicle, there are four neural networks associated, including one critic network, two actor networks, and an additional variation network which is used for policy coordination. The output of the critic network can be used to update the actor networks during training. For the variation network, even though the gradient cannot be backpropagated to update the actor–critic networks, the augmented reward function can guide the mobile vehicles to generate coordinated behaviors. In the network structures, FC means fully connected, and GRU is gated recurrent unit. GRU is used to extract information from the sequential observations. More details of the network parameters will be introduced in the experiment part. As the network parameters for each vehicle can be optimized in a decentralized way, this framework can be scalable to a large number of mobile vehicles.

**Algorithm** Now we formally present the algorithm in Algorithm 1 for an ego vehicle *i*. In this algorithm, we first initialize the parameters of the networks for the ego agent *i*. At each step, we generate the broadcast message $m_{i}$ based on the current observation $o_{i}$. The agent will then receive and aggregate messages from others and execute actions $a_{i}$. The tuples will be stored into the replay buffer D. During training, we sample a mini-batch of tuples from the buffer and perform gradient back propagation to update the critic network and actor networks. Finally, the variation network is also trained by maximizing the mutual information.   
**Algorithm 1:** Policy optimization for ego vehicle *i*
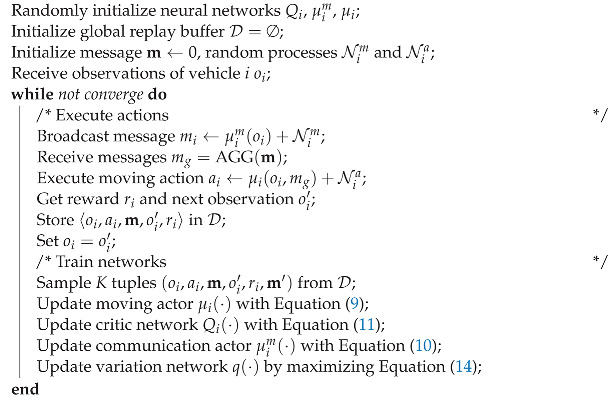


**Complexity** According to the above algorithm, we give a formal analysis of the time complexity of the training phase for each ego vehicle. At each step of training, the vehicle need to sample *K* tuples and update the networks. The update of the networks takes O(1) complexity for gradient descent. Suppose the convergence takes *C* steps. The time complexity of the algorithm will be O(KC). In the experiments, we will show that when choosing the batch size K=256, the algorithm takes about C=100,000 steps to converge. In fact, this algorithm can be computed on a cuda device very quickly. During execution, the policy can be computed in O(1) time.

### 5.2. Convergence Analysis

Given the above algorithm, in this section, we formally show that the value functions $Q_{i},i\in I$ can converge to an equilibrium point under certain assumptions:

**Assumption** **1.** *Every state s∈S and action $a_{i}\in A$, for i∈I, is visited infinitely often.*

**Assumption** **2.** *The critic learning rates $\alpha_{t}$ for optimizing Equation* (11) *satisfy $\sum_{\alpha_{t}=0}^{\infty }\alpha_{t}\left(s,a\right)=\infty$, and $\sum_{\alpha_{t}=0}^{\infty }{\left[\alpha_{t}\left(s,a\right)\right]}^{2}<\infty$ holds uniformly with probability 1.*

**Assumption** **3.** *The aggregated message $m_{g}$ is a representation of the global state information s and action a.*

**Assumption** **4.** *The stage game at each interval t has a global optimal point. The global points are selected by our algorithm to update the critic functions with probability 1.*

Assumptions 1 and 2 are weak ones that are easy to meet. Assumption 3 is met if (1) the communication message $m_{g}$ can encode the entire state without information loss; (2) every other vehicle’s policy can be inferred based on $m_{g}$. The two conditions are reasonable according to our communication-based framework. Assumption 4 is a strong assumption. It may not be easily met. However, our empirical experiments demonstrate that this assumption is satisfied mostly since the algorithm can converge in different scenarios. The convergence result mainly originates from the following lemma [40]:

**Lemma** **1.** *(**Szepesvari and Littman (1999), Corollary 5**)* Assume $\rho_{t}$ satisfies Assumption 2 and the mapping $P^{t}:Q\to Q$ has the following condition: there exists a number 0<γ<1 and a sequence $\lambda_{t}\geq 0$ converging to zero with probability 1 such that ${∥P^{t}Q-P^{t}Q^{*}∥}_{\infty }\leq \gamma {∥Q-Q^{*}∥}_{\infty }+\lambda_{t}$ for all Q∈Q and $Q^{*}=E\left[P^{t}Q^{*}\right]$, then the iteration defined by

(15) $$Q^{\prime }=\left(1-\rho_{t}\right)Q+\rho_{t}\left[P^{t}Q\right]$$

converges to $Q^{*}$ with probability 1.

According to Assumption 3, the messages $m_{g}$ is a compact representation of the global state *s* and actions a. Therefore, there is $Q_{i}\left(s,a\right)=Q_{i}\left(o_{i},a_{i},m_{g}\right)$. Define the transition function $P^{t}$ and the convergence point $Q^{*}$ as

**Definition** **1.** *Let $P^{t}:Q\to Q$ be a mapping on the complete metric space Q→Q, $P^{t}Q=\left(P^{t}Q_{1},P^{t}Q_{2},\ldots ,P^{t}Q_{N}\right)$, where*

(16) $$\begin{array}{c} P^{t}Q_{i}\left(s,a\right)=r_{i}+\gamma Q_{i}\left(o_{i}^{\prime },\mu_{i}\left(o_{i}^{\prime },\mu^{m}\left(s^{\prime }\right)\right),\mu^{m}\left(s^{\prime }\right)\right)) \end{array}$$

*for i∈I, where $\mu^{m}\left(\cdot \right)=\left(\mu_{1}^{m},\ldots ,\mu_{N}^{m}\right)$.*

**Definition** **2.** $Q^{*}$ is the convergence point if it satisfies

(17) $$\begin{array}{cc} Q_{i}^{*}(o_{i}, & o_{i},m_{g})=r_{i}+\gamma \sum_{s^{\prime }\in S}p\left(s^{\prime }|s,a\right)\\ \; & Q_{i}^{*}\left(o_{i}^{\prime },\mu_{i}\left(o_{i}^{\prime },\mu^{m}\left(s^{\prime }\right),\mu^{m}\left(s^{\prime }\right)\right)\right) \end{array}$$

With the above definitions, we show that the transition function $P^{t}$ is a “contraction mapping” with the fixed point at $Q^{*}$.

**Lemma** **2.** The convergence point is a fixed point: $E\left[P^{t}Q^{*}\right]=Q^{*}$.

**Proof.**  Since $Q^{*}$ is a convergence point in the game, the vehicles will still follow the current policy $\mu^{*}$. According to the Bellman equation (Equation (1)), there is

(18) $$\begin{array}{cc} \; & Q_{i}^{*}\left(o_{i},a_{i},m_{g}\right)\\ \; & =r_{i}+\gamma \sum_{s^{\prime }\in S}p\left(s^{\prime }|s,a\right)Q_{i}^{*}\left(o_{i}^{\prime },\mu^{m}\left(s^{\prime }\right),\mu_{i}\left(o_{i}^{\prime },\mu^{m}\left(s^{\prime }\right)\right)\right)\\ \; & =\sum_{s^{\prime }\in S}p\left(s^{\prime }|s,a\right)\left(\gamma Q_{i}^{*}\left(o_{i}^{\prime },\mu^{m}\left(s^{\prime }\right),\mu_{i}\left(o_{i}^{\prime },\mu^{m}\left(s^{\prime }\right)\right)\right)+r_{i}\right)\\ \; & =E[P^{t}Q_{i}^{*}\left(o_{i},x_{i},m_{g}\right)] \end{array}$$

where the forth line takes the expectation from $p(s^{\prime }|s,a)$ and the Bellman equation. □

Next, we show that $P^{t}$ is a “contraction mapping”. According to Assumption 3, there is $\mu_{i}\left(o_{i},m_{g}\right)=\mu_{i}\left(s\right)$. Similar to [41], the max-norm of the mapping operator can be defined as

$$\begin{array}{cc} \; & {∥Q-\hat{Q}∥}_{\infty }\equiv \max_{i}|Q_{i}-{\hat{Q}}_{i}|\\ \; & \equiv \max_{i,s}|\gamma Q_{i}\left(s\right)-\gamma {\hat{Q}}_{i}\left(s\right)|\\ \; & \equiv \max_{i,s,a}\gamma |Q_{i}\left(s,a\right)-{\hat{Q}}_{i}\left(s,a\right)| \end{array}$$

**Lemma** **3.** *${∥P^{t}Q-P^{t}\hat{Q}∥}_{\infty }\leq \gamma ∥Q-\hat{Q}∥\infty ,\;\forall Q,\hat{Q}\in Q$.*

**Proof.**  According to the transition function $P^{t}$, there is

(19) $$\begin{array}{cc} \; & {∥P^{t}Q-P^{t}\hat{Q}∥}_{\infty }\\ \; & =\max_{i,s}\gamma |Q_{i}\left(s,a\right)-{\hat{Q}}_{i}\left(s,a\right)|\\ \; & =\max_{i,s}\gamma |\prod_{j=1}^{N}\mu_{j}^{a}\left(s\right)Q_{i}\left(s\right)-\prod_{j=1}^{N}{\hat{\mu }}_{j}^{a}\left(s\right){\hat{Q}}_{i}\left(s\right)|\\ \; & \leq \max_{i,s}\gamma |\prod_{j=1}^{N}\mu_{j}^{a}\left(s\right)\left[Q_{i}\left(s\right)-{\hat{Q}}_{i}\left(s\right)\right]|\\ \; & \leq \max_{i,s}\gamma |Q_{i}\left(s\right)-{\hat{Q}}_{i}\left(s\right)|\\ \; & =\gamma {∥Q_{i}-{\hat{Q}}_{i}∥}_{\infty } \end{array}$$

The fourth line of equality comes from our Assumption 3 that the message $m_{g}$ is a compact representation of *s*. The fifth line of inequality is from Assumption 4 that the vehicles play the best response with respect to the broadcast message $m_{g}$. □

Summarizing the above two lemmas, it is proved that $P^{t}$ is a “contraction mapping” with the fixed point at $Q^{*}$. Thus, according to Lemma 1, there is the following.

**Theorem** **1.** Under Assumption 1-4, the sequence $\left(Q_{1},\ldots ,Q_{N}\right)$ updated by Algorithm 1 converges a fixed value $Q^{*}=\left(Q_{1}^{*},\ldots ,Q_{N}^{*}\right)$.

## 6. Evaluation

In this section, we first introduce the experiment setup, including the description of the environment, the baselines, and the model parameters. Next, we will show the performance of our algorithm with comparisons with other baselines. In particular, the results validate the importance of the consensual communication framework.

### 6.1. Experiment Setup

**Figure 4:**
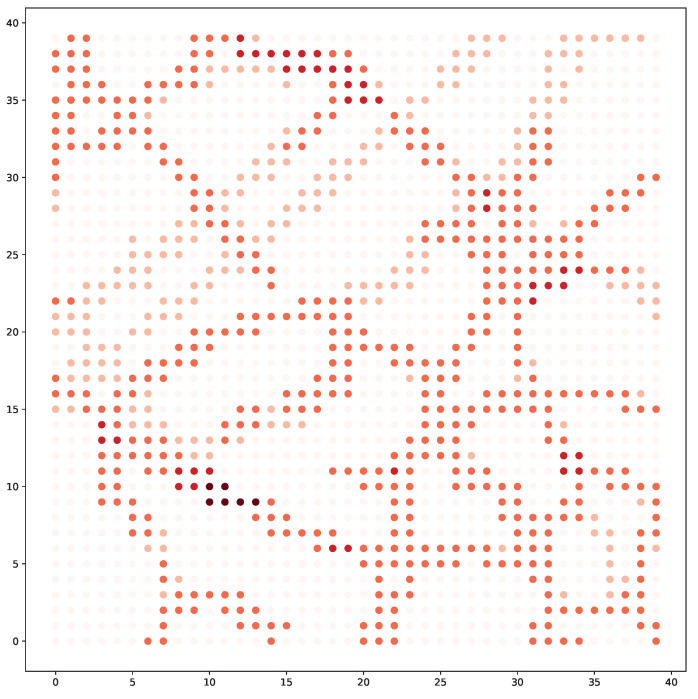
A snapshot of the event intensities in the target.

**The Environment** To validate the effectiveness of our algorithm, we manually construct a mobile sensing simulation environment based on real historical data set. The data set is collected from a road network from Google Map (Google Map: https://www.google.com/maps, accessed on 10 March 2022), which has the traffic volume at the road network across different hours (the data sets generated during the current study are available in the following https://www.dropbox.com/s/42cl68ns2fud5yk/GOOGLETraffic.zip?dl=0, accessed on 10 March 2022). We focus on an area of 10 km × 10 km square area centered at (48.16,16.33). In this map, we uniformly sample 40×40 points as the locations of events. For each position, the traffic volumes are extracted as the event intensities. An illustration of the event map at a given time is presented in Figure 4. The dots represent the events happening at different locations. The events have 5 levels of intensities as 0,1,2,3,4. We also add random uniform noise (0,1) to the event intensities for randomness. Dots with darker colors have higher event intensities. In this map, there assumed to be 5 charging stations at locations of (8,32), (32,8), (8,8), (32,32) and (20,20).

By default, we suppose the max speed of each vehicle is $S_{\max}=2$, and the sensing radius is $l_{i}=2$. Therefore, each vehicle can cover multiple events at the same time. The battery capacity of each vehicle is $b_{\max}=40$. During moving, the coefficient of battery consumption is β=1, $\Delta_{0}=1$. The vehicles can regularly navigate to the charging station, where they will be recharged $b_{0}=20$ units of battery at each time step. The penalty of running out of power is set as c=40. We will also try other values to validate the effectiveness of our algorithm. A small size of the replay buffer is set as $10^{5}$, since the vehicles policies may be dynamic.

**Baselines** We name our algorithm as ConComm (CONsensual COMMunication), and compare with the following baselines which can generate continuous actions.

- ConComm (no MI): In this algorithm, we implement the ConComm algorithm without the mutual information item. This comparison is to demonstrate the effectiveness of the mutual information item.
- DDPG [15]: In this algorithm, each mobile vehicle independently learns a policy to schedule the sensing path. The main drawback is that the multi-agent environment does not follow the Markov property, which may lead to the failure of this algorithm.
- MADDPG [9]: MADDPG uses the CTDE framework, where there is a global critic function that has access to the historical samples from all mobile vehicles. However, the policies of the vehicles are not coordinated explicitly during execution.
- MAPPO [42]: This algorithm is a multi-agent version of PPO. It has achieved state-of-the-art performance in many scenarios.

**Model Parameters** For different algorithms, we use similar critic network structures with an FC layer with 64 hidden units. The FC layer is followed by a ReLU activation layer for non-linear activation. The output is connected with a GRU layer with 64 hidden units and then fed into another FC layer to output the critic value. The actor networks have a similar structure. The only difference is the output of the networks. The communication actor network outputs a message with size 6 followed by a sigmoid layer to restrict the message in the range (0,1). The messages are aggregated with a MEAN operator, i.e., $m_{g}=\frac{1}{N}\sum_{i\in I}m_{i}$. The moving actor network outputs a vector of size 2, followed by a sigmoid layer to restrict the range of the speed and angle. Maximum speed and angle are used to project the outputs into new ranges. For the variation network, the input is the embedding after the FC layer. It is then fed into two FC layers with 64 hidden units to predict the aggregate message. Mean squared error is used as the loss function for the variation network. The weight of the MI item ρ is set as 0.5 so that different parts of the reward function are comparable.

### 6.2. Performance Analysis

**Figure 5:**
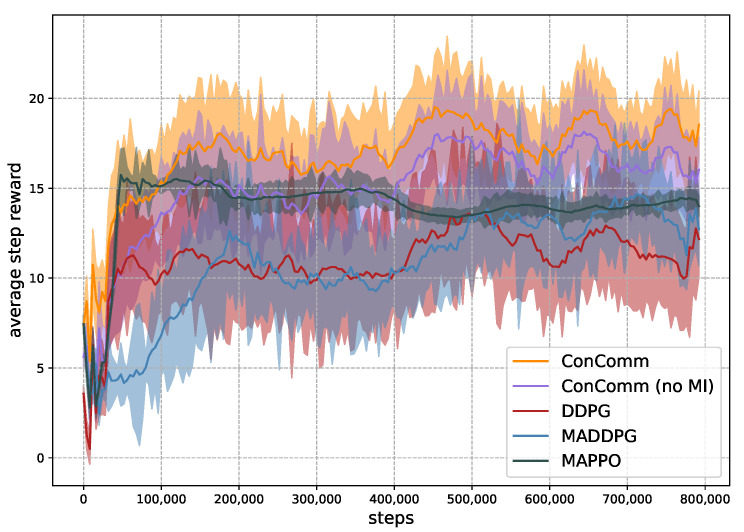
The performance of different algorithms in the execution phase.

**Convergence of Training** In the first experiment, we assume there are N=12 mobile vehicles, and examine the convergence of the algorithms during training in Figure 5. The average step reward is evaluated every 200 steps. We assume different vehicles share the same network parameters. Nonetheless, the vehicles can still behave differently with local observations. The y-axis represents the average step reward for each vehicle $r=\frac{1}{N}\sum_{i\in I}r_{i}$. Each of the RL-based algorithms is trained 3 times. The shaded area represents one standard deviation. As presented, our proposed ConComm achieves the highest performance at most of the time. The average step reward of ConComm can converge to around 17 after about only 50,000 steps. The performance then stabilizes around at this level. Moreover, the variance of ConComm is also more stable compared to others. This is because the vehicles are more likely to have coordinated behaviors. ConComm (no MI) is the algorithm without explicit policy coordination. The result can be relatively high due to the communication among the mobile vehicles. However, the performance is worse than ConComm, which validates the effectiveness of the MI item. DDPG has the worst performance among the algorithms. This is mainly due to the fact that the vehicles make decisions independently. Therefore, there may be lots of repeated sensing efforts among the vehicles. MADDPG and MAPPO have similar performances that are slightly better than DDPG. The main reason is that they adopt the “centralized training, decentralized execution” mechanism. However, in the execution phase, there may still be uncoordinated behaviors with unseen environment states. Different vehicles may not achieve consensus before making decisions. The above comparisons show that communication plays an important role in coordinating the vehicles’ behaviors.

**Figure 6:**
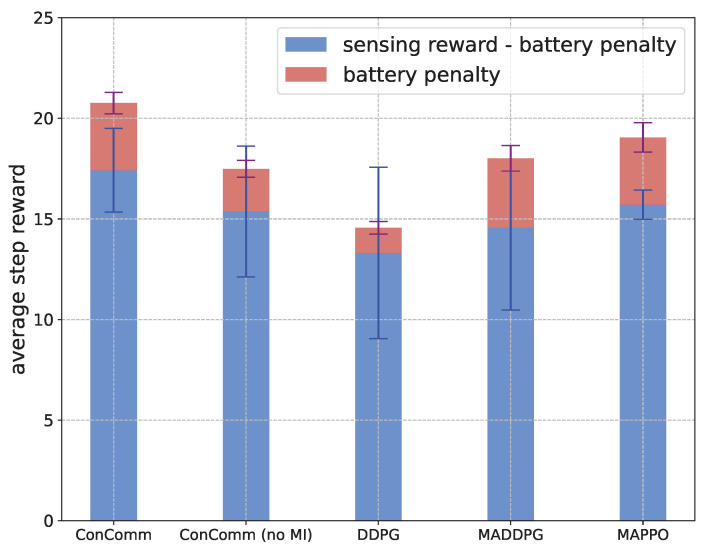
The performance of different algorithms in the execution phase.

**Performance during Execution** After training, we fix the network parameters and compare the performance of different algorithms in the simulation environment without exploration. The results are shown in Figure 6. In this figure, the height of each bar represents the sensing reward, where the red part is the battery penalty, and the blue part is the true average reward, which equals the sensing reward minus the battery penalty. The algorithm with the highest blue bar has the best performance.

As presented, our proposed ConComm achieves the best performance (the blue part) among the algorithms. In particular, the sensing reward (the blue+red part) also outperforms other algorithms significantly. This is because the vehicles in ConComm can avoid repeated sensing through communication. The ConComm (no MI) can also have high performance. It achieves lower battery penalty because the vehicles’ behaviors will not be affected by the communication messages explicitly. DDPG also performs well in charging since each vehicle only cares about its own reward. However, the global sensing reward can be quite limited, which may be caused by the lack of coordination. For the MADDPG and MAPPO algorithms, as they lack the mechanism of coordination in the execution phase, they may not perform as well as our ConComm algorithm. In summary, to achieve high performance, the vehicles should not only try to sense more events with the limited battery, but they need also coordinate with others to avoid repeated sensing.

**Figure 7:**
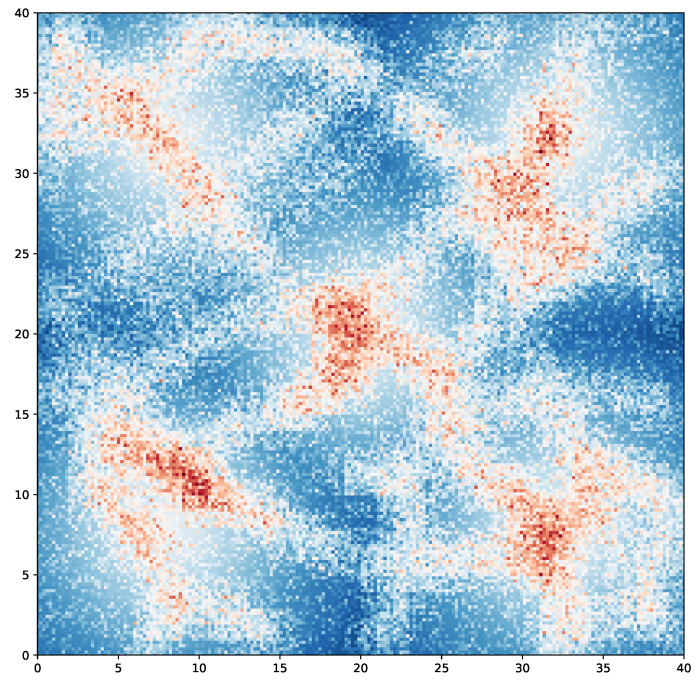
The heatmap of the vehicles trajectories of ConComm.

We also investigate the trajectories of the vehicles in our ConComm to show the effectiveness. We collect the vehicles’ trajectory in the execution phase for 1000 steps and obtain the appearance count in the map. The appearance counts are normalized and plotted as a heatmap. The result is presented in Figure 7. In the heatmap, areas with a redder color are visited more often by the mobile vehicles, and the blue areas are visited less often. Compared with Figure 4, the areas where the event intensities are higher also have more vehicle appearances. These areas are dispersed since the vehicles can cooperate to maximize the coverage and reduce repeated sensing. Moreover, the areas near the charging stations also have redder colors; this is because the vehicles regularly moves to the stations for charging. Above all, the heatmap validates that the vehicles of ConComm can not only navigate back for charging, but also properly move to the areas with high event intensities. This heatmap illustrates that our proposed ConComm can properly coordinate the navigation of the vehicles.

**Policy Coordination via Communication** The above two experiments have already shown that explicitly coordinating the policies of different mobile vehicles is crucial for cooperative sensing. In this part, we investigate the effect of coordination by adjusting the weight of the MI item. In addition to the default value ρ=0.5, we change the weight ρ to different values from 0 and 1 and observe the convergence process during training. Note that when ρ is 0, the algorithm degrades to the case of ConComm (no MI). When ρ is 1, the vehicles neglect the sensing reward and battery penalty, and focus only on coordinating with others.

The results are shown in Table 2. As presented, introducing the policy coordination can significantly improve the performance when ρ is non-zero. This validates that positive communication is necessary for coordinating the decentralized vehicles. Meanwhile, when the coefficient is too large, the performance may decrease since the vehicles care more about coordination and less about sensing reward. When the coefficient reaches 1, the vehicles focus only on the coordination and thus the sensing reward is very poor. The results show that the vehicles need to balance between coordination and sensing. The performance will degrade if focusing on only one of them.

**Validating the Variation Network** In this part, we show that the communication message indeed influences the vehicles’ moving policy. More concretely, we compute the cross entropy between the policy $\mu_{i}$ and the neighbors’ aggregated message $m_{g}$ as $H(\mu_{i},m_{g})$. The policy $\mu_{i}$ is represented as the historical trajectories $traj_{i}$. Cross entropy measures the average number of bits needed to identify an event drawn from the set if a coding scheme used for the set is optimized for an estimated probability distribution, rather than the true distribution. It can also be regarded as the distance between the two probability distributions. A low cross entropy distance indicates that the two probability distributions could have high correlation.

**Figure 8:**
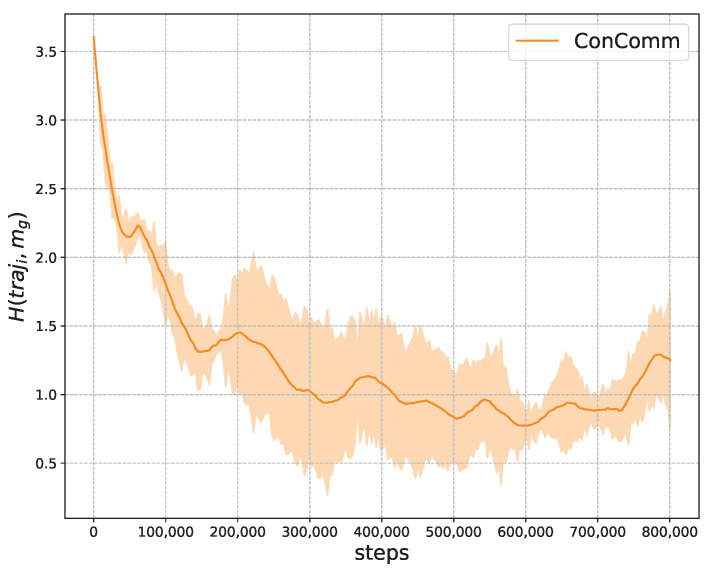
The cross entropy between the vehicles aggregated message $m_{g}$ and moving policy $\mu_{i}$ represented as $traj_{i}$. The value is lower when the message is more correlated with the vehicles’ moving policies.

We present the dynamics of the cross entropy during training in Figure 8. As presented, the cross entropy is high at the beginning. This is because the vehicles have not learned to correlate with the communication message. As the training proceeds, the cross entropy value becomes lower and stabilizes at about 1.0. This validates that the policy $\mu_{i}$ becomes more correlated with the communication.

**Figure 9:**
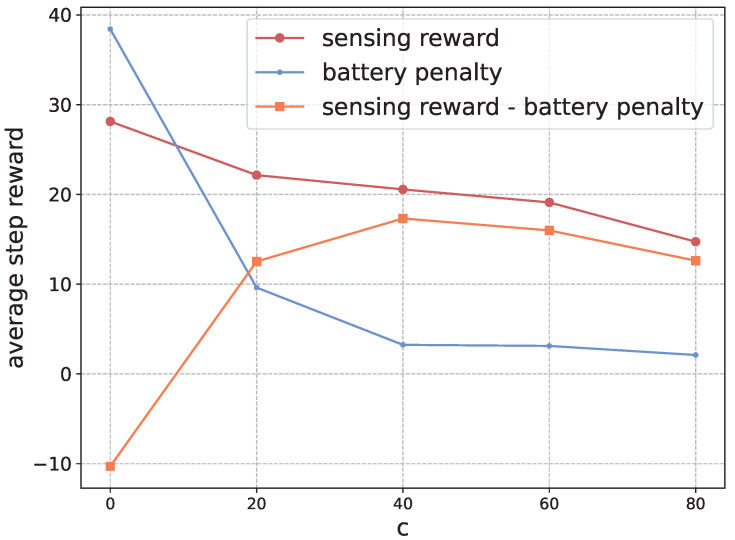
The average step reward (orange part), sensing reward (red part) and battery penalty (blue part) in the execution phase with respect to different values of *c*.

**Validation the Penalty of Battery** Next, we investigate the effect of the hyper-parameter *c* in shaping the battery penalty. Generally, with a larger value of *c*, the vehicles will navigate to the charging station more frequently to avoid running out of battery. In practice, this parameter can be set freely by the vehicles and our algorithm can adapt to different values of *c*. In this experiment, we train the algorithms with different values of *c* and validate the performance with the default value c=40. Figure 9 presents the results.

As presented, when *c* is 0, the vehicles will not care about the battery penalty and focus only on sensing events. Therefore, there will be high sensing reward, but the battery penalty will also be very high, leading to low average step reward. When the value of *c* increases, the vehicles will be more conservative to run out of power. They will have low battery penalty. However, the sensing reward will also decrease. In general, choosing a proper value of *c* can balance the preference of sensing and battery. In practice, we can set the value of *c* as the cost of reclaiming the vehicles when they run out of power. If this is unacceptable, we can also enforce the vehicles to navigate back to the charging station if needed.

**Figure 10:**
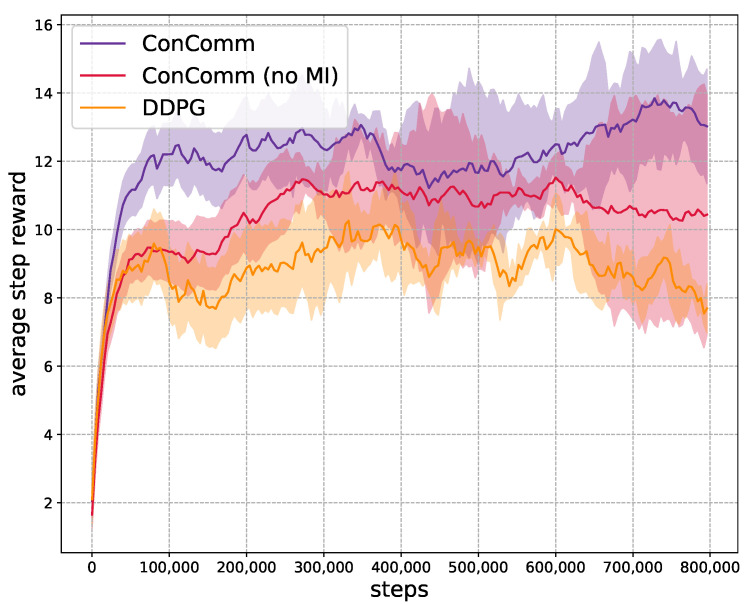
The scalability.

**Scalability** In this last experiment, we validate the scalability of ConComm. We increase the number of mobile vehicles to 128 and charging stations to 16. The map is divided into 80×80 grid space with charging stations randomly and uniformly distributed. Similar to that above, we assume the mobile agents share the same network parameters. The algorithms of MADDPG and MAPPO would take too much time, so we only present the result of ConComm, ConComm (no MI) and DDPG. As shown in Figure 10, the ConComm algorithm can still achieve better performance. When there are more agents, they may become more easy to coincide. So the average step reward will be lower than before. Nonetheless, ConComm can still successfully coordinate the behaviors of the agents and achieve high performance. In this case, as there is no explicit coordination, the variance of ConComm (no MI) will be larger. The result of DDPG is also not stable since the vehicles’ policies are mostly dynamic, leading to low efficiency of coordination. Moreover, the performance of DDPG will even degrade after about 600,000 steps. This may result in the DDPG agents being not coordinated and falling into local optimal points.

## 7. Conclusions

This paper studies the problem of mobile sensing in an open, dynamic environment. To maximize the long-term spatial–temporal coverage of the events, we propose a decentralized policy coordination framework. The main idea is to introduce a communication mechanism among the mobile vehicles. On one hand, the vehicles can share local information with each other to break through the dilemma of decentralized execution; on the other hand, the vehicles can have coordinated behavior with enforced positive communication. In particular, the consensual communication is achieved by maximizing the mutual information between the received message and the policy. We conduct extensive experiments to validate the performance of our algorithm. The results show that our algorithm can converge very fast in the training phase, and outperforms other baselines significantly in the execution phase. Moreover, the experiments show that the consensual communication mechanism plays an important role in coordinating the behaviors.

For future works, we aim to extend the current method from two aspects. First, the battery constraints in this paper are relaxed as part of the objective, and may lead to violations. Therefore, we need to devise method with “hard” constraints. Second, we will improve the interpretability of the communication messages to understand the internal mechanism that promotes the cooperation among the vehicles.

## Figures and Tables

**Table 1:** Key parameter table of system model.

Notation	Definition
I	the set of mobile vehicles: I={1,2,…,N}
E	the set of events
*t*	the time step t∈{0,1,…,∞}
$\left(x_{i}^{t},y_{i}^{t}\right)$	the position of vehicle *i* at step *t*
$\left(\nu_{i}^{t},\phi_{i}^{t}\right)$	moving speed and angle of vehicle *i* at step *t*
$b_{i}^{t}$	battery capacity of vehicle *i* at step *t*
$\Delta_{i}^{t}$	battery consumption rate of vehicle *i* at step *t*
$b_{0}$	battery charging rate at the charging station
$l_{i}$	the sensing radius of vehicle *i*
$\tau_{e}^{t}$	the event intensity of event e∈E at step *t*
*c*	the penalty for running out of battery
$r_{i}^{t}$	the reward of vehicle *i* at step *t*
$\mu_{i}\left(\cdot \right)$	the moving policy function of vehicle *i*
$\mu_{i}^{m}\left(\cdot \right)$	the communication policy function of vehicle *i*
$Q_{i}\left(\cdot \right)$	the action–value function of vehicle *i*
$v_{i}\left(\cdot \right)$	the state value function of vehicle *i*
q(·)	proxy for the posterior function
I(·)	mutual information function
ρ	the weight of the MI reward

**Table 2:** Different parts of the performance of ConComm. The average step reward can be described as the difference between the sensing reward and the battery penalty.

ρ	Sensing Reward	Battery Penalty	Average Step Reward
0	17.33	2.14	15.19
0.25	** 20.83 **	3.20	** 17.35 **
0.5	20.76	3.34	17.42
0.25	19.58	4.20	15.38
1	7.87	37.36	−29.49

## Data Availability

The traffic data address: https://www.dropbox.com/s/42cl68ns2fud5yk/GOOGLETraffic.zip?dl=0 (accessed on 23 October 2022).
